# Final Fusion Strategies in Early-Onset Scoliosis: Does Implant Density Make a Difference After Magnetically Controlled Growing Rod Treatment?

**DOI:** 10.3390/children12060731

**Published:** 2025-05-31

**Authors:** Paolo Brigato, Leonardo Oggiano, Sergio De Salvatore, Davide Palombi, Sergio Sessa, Umile Giuseppe Longo, Andrea Vescio, Pier Francesco Costici

**Affiliations:** 1 Research Unit of Orthopaedic and Trauma Surgery, Department of Medicine and Surgery, Università Campus Bio-Medico di Roma, Via Alvaro del Portillo, 21-00128 Rome, Italy; paolo.brigato@unicampus.it (P.B.); sergio.desalvatore@gmail.com (S.D.S.); g.longo@policlinicocampus.it (U.G.L.); 2 Fondazione Policlinico Universitario Campus Bio-Medico, Via Alvaro del Portillo, 200-00128 Roma, Italy; 3 Orthopedic Unit, Department of Surgery, Bambino Gesù Children’s Hospital, 00050 Rome, Italysergio.sessa@opbg.net (S.S.); pierfrancesco.costici@opbg.net (P.F.C.); 4 Department of Pediatric Neurosurgery, Fondazione Policlinico Agostino Gemelli IRCCS, Università Cattolica del Sacro Cuore, 00168 Rome, Italy; 5 Department of Life Science, Health, and Health Professions, Link Campus University, Via del Casale di San Pio V, 44-00165 Rome, Italy; a.vescio@unilink.it; 6 Department of Orthopaedic and Trauma Surgery, “Mater Domini” University Hospital, “Magna Græcia” University, 88100 Catanzaro, Italy

**Keywords:** early-onset scoliosis, magnetically controlled growing rod, MCGR, posterior spinal fusion, pedicle screws, implant density, MAGEC, high density, low density

## Abstract

Background/Objectives: Early-onset scoliosis (EOS) frequently requires growth-friendly interventions, such as magnetically controlled growing rods (MCGRs), followed by definitive spinal fusion upon skeletal maturity. The optimal implant density (ID) for final posterior spinal fusion in these patients remains controversial. This study aimed to compare the radiographic, surgical, and economic outcomes associated with high-density (HD) versus low-density (LD) screw constructs in EOS patients previously treated with MCGRs undergoing definitive fusion. Methods: This retrospective study included 27 EOS patients who underwent definitive posterior spinal fusion between January 2017 and September 2022. Participants were categorized into two groups: HD (n = 13) and LD (n = 14). Primary outcomes included coronal and sagittal radiographic parameters assessed at early postoperative and final follow-up visits (minimum of 2 years). The secondary outcomes analyzed were major postoperative complications (grade ≥ IIIB according to Clavien–Dindo–Sink Classification [CDSC]), operative time, blood loss, hospital stay length, and total implant costs. Results: Baseline characteristics between the HD and LD groups were comparable. Early postoperative radiographic assessment demonstrated significantly greater thoracic kyphosis (16.3 ± 7.6° vs. 10.9 ± 14.4°, *p* = 0.021) and T1-S1 spinal height (43.3 ± 6.7 mm vs. 39.1 ± 4.3 mm, *p* = 0.039) in the HD group. At final follow-up, only T1-S1 spinal height remained significantly higher in the HD group (45.4 ± 7 mm vs. 39.7 ± 5.1 mm, *p* = 0.021). Implant costs were significantly higher in the HD group (EUR 6046.5 ± 1146.9 vs. EUR 4376.4 ± 999.4, *p* < 0.001), while operative time, blood loss, and hospital stay length showed no significant differences. HD constructs had three major complications requiring surgical revision, whereas LD constructs reported no perioperative complications but experienced three late-onset complications also necessitating revision surgery. Conclusions: LD constructs provided comparable long-term radiographic and clinical outcomes to HD constructs, with significantly lower implant-related costs. Despite initial superior kyphosis correction in HD constructs, this benefit diminished by the final follow-up. These findings support a selective, lower-density screw placement strategy to minimize costs and surgical complexity without compromising patient outcomes in EOS undergoing definitive spinal fusion.

## 1. Introduction

Early-onset scoliosis (EOS) is a complex and heterogeneous condition defined by a spinal deformity before age 10 [1]. It encompasses many etiologies, including idiopathic, congenital, syndromic, and neuromuscular forms, often with significant cardiopulmonary implications [2]. In recent years, non-traditional distraction-based systems such as magnetically controlled growing rods (MCGRs) have revolutionized the treatment of EOS, allowing for progressive, non-invasive spinal lengthening during growth while delaying definitive spinal fusion. In contrast, the dual-rod technique remains the gold standard for final fusion, even if it carries out complications and re-intervention risks [3,4,5,6].

At skeletal maturity, however, a final fusion is generally required to stabilize the spine and prevent future curve progression [7,8,9]. In this stage, the optimal surgical strategy, particularly regarding the number and distribution of pedicle screws, remains controversial. While high-density (HD) constructs are believed to provide stronger fixation and potentially more significant correction, they are associated with increased surgical time, blood loss, risk of complications, and significantly higher healthcare costs [10,11,12]. In contrast, low-density (LD) constructs have demonstrated comparable radiographic and clinical outcomes in Adolescent Idiopathic Scoliosis (AIS), mainly when screws are strategically distributed rather than uniformly placed [13,14,15].

Despite the increasing body of evidence in AIS surgery, there is a lack of data regarding the characteristics and outcomes of implant density (ID) in the EOS population, particularly in patients undergoing final fusion after MCGR treatment [16,17,18,19,20]. This gap in the literature exists partly because there are currently no studies specifically evaluating graduation surgery using only pedicle screws in EOS patients treated with MCGRs, with most studies primarily reporting results based on the use of hybrid constructs. Consequently, considering the distinct biomechanical properties of EOS bones, which may influence the strength and distribution of fixation, there is an urgent need for studies that assess homogeneous constructs composed exclusively of pedicle screws in post-graduation surgery outcomes [4,15].

To the authors’ knowledge, no prior study has specifically investigated the radiographic and surgical outcomes of all-screw constructs in graduated patients following treatment with MCGRs, with particular attention to ID. Therefore, this study aims to assess the impact of ID on definitive posterior spinal fusion in patients with early-onset scoliosis previously managed with the MCGR system. Specifically, the study compares high-density and low-density constructs in terms of radiographic correction, complication rates, and economic implications.

## 2. Materials and Methods

### 2.1. Study Population

This retrospective study analyzed ambulatory pediatric patients diagnosed with idiopathic, neuromuscular, or syndromic EOS [21]. All patients included had completed an entire course of treatment using the MAGEC^®^ system (MAGnetic Expansion Control, NuVasive Specialized Orthopedics, San Diego, CA, USA) and subsequently underwent elective final fusion surgery at a single, high-volume spinal surgery center between January 2017 and September 2022. The earliest MAGEC^®^ device (Nuvasive Specialised Orthopaedics, San Diego, CA, USA) implantation in this cohort was in July 2011, with patients undergoing scheduled distractions every three months as part of the standard lengthening protocol. Only patients who had been treated exclusively with the MAGEC^®^ system, without the use of alternative distraction techniques, were included. Before undergoing definitive spinal fusion, all patients completed thorough cardiopulmonary evaluations and were medically cleared for surgery.

The following exclusion criteria were applied to patients:

Non-walking;
Congenital EOS;
Without final fusion after completing the lengthening program;
With hybrid constructs (HCs), consisting of instrumentation with screws, hooks, and/or universal clamps, after the final fusion.

### 2.2. Study Design

The results in terms of radiographic and surgical outcomes are compared between a high screw density group (HD group) and a low screw density group (LD group). The patients were consecutively enrolled in the database. The operating surgeon decided to use an HD or LD construct based on clinical judgment and surgical experience. Several patient-specific factors were considered, including ambulatory status, presence of osteoporosis, pedicle integrity, spinal rigidity, and duration of prior treatment. These parameters helped guide the choice of implant density to optimize construct stability while minimizing surgical risks and complications. Clinical evaluations and radiological measurements, conducted by an experienced radiologist using bi-planar radiographs, were performed before final fusion (after the lengthening program, t1), at discharge after vertebral arthrodesis (t2), and at the last follow-up (FUP) (t3). A minimum FUP period of 2 years after the final fusion was required for a patient to be included in the study.

This study was conducted according to the Strengthening the Reporting of Observational Studies in Epidemiology (STROBE) guidelines [22].

### 2.3. Outcomes

The primary outcome of this study was the assessment of frontal plane (main curve, frontal balance, shoulder balance, pelvic balance) and sagittal plane parameters (SVA, LL, TK, CL, SS, T1-T12, and T1-S1 lengths) both in the early postoperative period and at the final follow-up, compared to preoperative values, with a focus on differences between the HD and LD screw groups [23,24]. Secondary outcomes included the incidence of major postoperative complications, operative time, intraoperative blood loss, and length of hospital stay.

### 2.4. Study Variables and Outcome Measures

Patient medical records were reviewed to collect demographic, surgical, radiological, and instrumentation-related data about the final fusion procedure. The collection of postoperative clinical data was conducted retrospectively through the review and analysis of patient files, which contained detailed medical records and treatment histories. All radiographic, clinical, and surgical variables included in this study were defined and measured according to standardized parameters [23,24]. Table 1 summarizes the study variables.

ID was assessed by calculating the total number of fused spinal levels and the corresponding number of pedicle screws used in each case. These values were utilized to derive the average screw density per level using the following formula:

ID = Total Number of Screws/Number of Fused Levels

This parameter was recorded for all patients included in the study, yielding a cumulative mean ID of 1.11. Patients with an average screw density below 1.11 were assigned to the LD group, whereas those with a density equal to or greater than 1.11 were assigned to the HD group. The current literature does not provide a standardized or universally accepted threshold for distinguishing between LD and HD screw constructs in spinal fusion surgery, which remains an area of ongoing debate and research. In the present study, the cutoff value of 1.11 screws per fused level was established based on the specific distribution characteristics of the analyzed patient cohort. This threshold was selected with the intent of facilitating an internal comparison between the groups and ensuring a consistent framework for evaluating ID within this specific study population. However, it is important to note that this value should not be interpreted as a universally applicable or generalizable standard. Rather, it reflects the particular characteristics, demographics, and clinical circumstances of the cohort under investigation. As such, the cutoff value may not be directly applicable to other patient populations, cohorts, or clinical settings, where differing anatomical, biomechanical, and surgical factors could necessitate alternative thresholds or approaches. Major perioperative complications were classified according to the Modified Clavien–Dindo–Sink Classification (CDSC) for EOS patients as Grade ≥ IIIB [25].

Regarding postoperative surgical data, the following parameters were evaluated:

Postoperative hemoglobin (Hb) levels were determined by comparing the first blood test results obtained after surgery with those collected immediately before the procedure.

Operative time was measured from the initial skin incision to the completion of the immediate postoperative radiograph. This data was collected retrospectively through the consultation of the official operative report, which is written by the primary surgeon at the end of each surgical procedure.

Length of hospital stay (LOS) was defined as the total duration of hospitalization, calculated from the day of admission to the day of discharge.

### 2.5. Surgical Procedures

The same experienced surgical team performed all surgical procedures, with patients placed in the prone position on a radiolucent carbon operating table under general anesthesia. Intraoperative neurophysiological monitoring, including motor-evoked potentials (MEPs) and sensory-evoked potentials (SEPs), was employed throughout the procedure to ensure spinal cord integrity. A midline incision was made following the previous surgical scar, and subperiosteal dissection of the paraspinal musculature was performed to expose the posterior elements of the spine. The MCGRs and any hybrid fixation devices, such as hooks or universal clamps, were removed. In cases where pedicle screws had been used in the initial construct, they were either retained or exchanged for larger screws, depending on intraoperative assessment. Additional pedicle screws were then inserted into the remaining intact vertebrae using the free-hand technique. When indicated, posterior column osteotomies (PCOs), grade I or II according to Schwab’s classification, were performed at the curve apex to enhance deformity correction [26,27]. Screw positioning was verified with fluoroscopy before connecting two cobalt-chromium rods and executing corrective maneuvers. Local autograft bone harvested from the spinous processes was used to facilitate fusion. Postoperative anteroposterior radiographs were obtained immediately after surgery. Patients were initially admitted to the Intensive Care Unit (ICU) for monitoring on the first postoperative day and, if clinically stable, were transferred to the Orthopedic Department for ongoing recovery.

### 2.6. Statistical Analysis and Ethics

SPSS Statistics 25.0 v (IBM, Armonk, NY, USA) was used for statistical analysis. Continuous variables were reported as means with standard deviations or medians with interquartile ranges, depending on data distribution. The Mann–Whitney U test was employed to compare differences between two independent groups when the data did not meet the assumptions of normality. The normality of data distribution was evaluated using the Shapiro–Wilk test. A *p*-value of less than 0.05 was considered statistically significant.

This study was conducted according to international ethical standards for clinical research, as outlined in the Helsinki and Istanbul Declarations. In line with local institutional review board regulations, ethical approval for retrospective studies was not required, as the data were anonymized before analysis.

## 3. Results

### 3.1. Patient Selection

**Figure 1:**
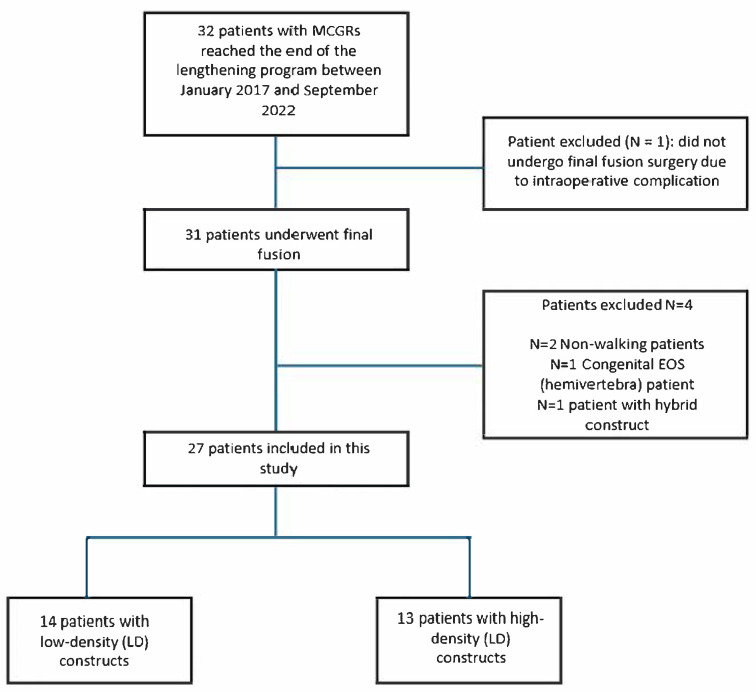
Patient selection.

Thirty-two patients completed the lengthening program between January 2017 and September 2022. One patient was excluded from the study as graduation surgery was not performed due to a potentially life-threatening intraoperative complication, leading to the removal of the MCGRs without proceeding to final fusion. Four additional exclusions were made among the 31 patients who underwent final fusion: 2 patients were non-ambulatory, 1 patient had congenital early-onset scoliosis (specifically a T4 hemivertebra), and 1 patient was treated with a hybrid construct. Consequently, the study included 27 patients, who were categorized based on implant density into two groups: 14 patients in the LD group (<1.11) and 13 patients in the HD group (≥1.11). The patient selection process is outlined in Figure 1.

### 3.2. Patient Population

Among the 27 patients, 20 were female (74.1%) and 7 were male (25.9). The most prevalent etiology in the HD group was idiopathic EOS (nine patients, 69.2%), while in the LD group, syndromic EOS was more common (eight patients, 57.1%). Both groups predominantly had thoracic curves, with 10 patients (76.9%) in the high-density group and 7 patients (50%) in the low-density group. However, the low-density group had a higher proportion of double curves (six patients, 42.8%) than the high-density group (two patients, 15.4%). The groups were similar in terms of main curve side and lumbar modifiers. Table 2 resumes the baseline characteristics of the study groups.

### 3.3. Demographic Characteristics

Table 3 summarizes the demographic characteristics of patients before final fusion. The average age at the time of surgery was 14.3 ± 1.4 years (range: 11–17), and the average BMI was 21.9 ± 5.8 kg/m^2^ (range: 12.4–36.5) for the entire cohort. The total FUP time from MCGR surgery to final fusion was 60.1 ± 25 months, and the FUP time after final fusion was 43.6 ± 23.7 months in the HD group. In the LD group, the total FUP time from MCGR surgery to final fusion was 66.5 ± 29.6 months, and the FUP after final fusion was 33.6 ± 12.4 months. There were no significant differences between the groups (*p* > 0.05).

### 3.4. Fixation and Implant Characteristics

The mean number of screws used per procedure, the number of fused levels, and the number of pedicle screws per level differed significantly between the HD and LD groups (*p* < 0.05). In the HD group, six patients (46.1%) underwent at least one PCO, compared to one patient (7.1%) in the LD group. The choice of Upper Instrumented Vertebra (UIV) and Lower Instrumented Vertebra (LIV) was similar between the two groups. However, the LD group exhibited a more extended fusion, with LIV at L4 in eight patients (57.1%) compared to four patients (30.8%) in the HD group, which was attributable to the characteristics of the spinal curves. Table 4 and Table 5 provide a summary of the fixation and osteotomy characteristics.

### 3.5. Coronal and Sagittal Plane Characteristics

Table 6 and Table 7 report the coronal and sagittal alignment parameters assessed at three time points: end of lengthening (t1), immediately after final fusion (t2), and at final follow-up (t3). At T1, the two groups were found to be comparable in terms of radiographic measurements, indicating no significant differences in the baseline spinal parameters before the intervention, except for a statistically significant difference observed only in Lumbar Lordosis (LL), with higher values recorded in the HD group compared to the LD group (56.2 ± 14.2° vs. 42.8 ± 14.9°, *p* = 0.023). At t2, the HD group demonstrated significantly greater thoracic kyphosis (TK) (16.3 ± 7.6° vs. 10.9 ± 14.4°, *p* = 0.021) and T1-S1 spinal height (43.3 ± 6.7 mm vs. 39.1 ± 4.3 mm, *p* = 0.039). By t3, T1-S1 height remained significantly higher in the HD group (45.4 ± 7 mm vs. 39.7 ± 5.1 mm, *p* = 0.021), while no other significant differences were found between groups.

### 3.6. Implant Cost and Surgical Outcomes

Table 8 presents a summary of final fusion surgical outcomes. The total cost of the implant was significantly higher in the HD group compared to the LD group (EUR 6046.5 ± 1146.9 vs. EUR 4376.4 ± 999.4, *p* < 0.001). No statistically significant differences were found regarding surgery duration, blood and hb loss, and LOS (*p* > 0.05).

### 3.7. Postoperative Complications

Table 9 summarizes the data on perioperative complications, classified according to the CDSC and late-onset complications after final fusion. Both groups experienced a total of three complications (23.1% of patients in the HD group vs. 21.4% in the LD group). Two of the three complications in the HD group occurred in the perioperative period, accounting for a deep surgical site infection (SSI) and a cerebrospinal fluid (CSF) leak, which were both classified as major according to CDSC. The last complication was a rod rupture, which required revision surgery. The LD group had no perioperative complications but developed two distal junctional kyphosis (DJK) and one delayed SSI during the 2-year follow-up, all of which required surgical revision.

## 4. Discussion

The present study explores the role of ID in final spinal fusion after MCGR treatment in EOS. This study’s results demonstrate that HD constructs significantly increased implant costs, more significant T1-S1 length gains with early TK improvement, with no other significant differences in radiographic outcomes or complication rates compared to low-density (LD) constructs.

These findings are consistent with several recent AIS population studies [10,18]. Luo et al. performed a pooled analysis of 827 AIS patients. They found no significant difference between the HD and LD groups in major curve correction, thoracic kyphosis, or quality of life scores during the first posterior fusion surgery [15]. However, LD constructs were associated with significantly reduced operative time, blood loss, and hospital charges [14]. In particular, in terms of cost savings, Larson et al. estimated a potential annual cost saving of USD 11–20 million in the US by adopting lower screw density strategies without compromising outcomes [10,11].

Despite this growing literature in AIS, specific data on ID in EOS patients treated with MCGRs remain extremely limited [4,12,13]. No large-scale or prospective study has specifically compared LD and HD strategies in the context of final fusion after MCGR distraction, representing a knowledge gap in the field.

Unlike AIS, the spine in EOS is frequently altered by spontaneous auto-fusion and mechanical stress [28]. Menapace et al. addressed a key consideration in the EOS population regarding this phenomenon [28]. In their series of 14 EOS patients treated with MCGRs and final fusion, auto-fusion was present in up to 78% of cases at the time of definitive surgery, most commonly at the proximal anchors. This finding suggests that the biomechanical demands during final fusion may be lower than assumed, particularly in patients with stiff, partially fused segments [28].

In this series, postoperative TK initially improved more in the HD group. Immediately after final fusion, HD patients had a mean TK of 16.3°, compared to 10.9° in LD patients (*p* = 0.021), indicating a more rapid restoration of sagittal alignment in the early postoperative period. However, this advantage diminished over time. At the last follow-up, TK values were nearly equivalent between the groups—15.8° in HD vs. 13.7° in LD (*p* = 0.293). These findings suggest that while HD constructs may offer an initial kyphosis correction advantage, their long-term sagittal alignment is not superior to that of LD constructs.

In terms of general outcomes and complications, Mainard et al., in a cohort of 66 EOS patients post-MCGR treatment, reported an implant-related complication rate of 29%, with the final correction rate (major coronal angle reduced from 62° to 28°) not directly correlated with the number of screws used [29]. Their study did not stratify patients by ID, but the variability in instrumentation patterns suggests that surgeons are already adapting density according to intraoperative findings [29].

Regarding complications, our results echo the findings of Thakar et al., who, in a systematic review of 336 MCGR patients, reported a 44.5% complication rate and a 33% unplanned revision rate [4]. The most common issues were anchor pullout (11.8%), implant failure (11.7%), and rod breakage (10.6%). Interestingly, implant failure and rod breakage were more common in single-rod constructs, reinforcing the need for dual-rod stability but not necessarily for high ID [4].

Interestingly, in this cohort, while the overall complication rate was similar between the HD and LD groups, early perioperative complications were more frequent in the HD cohort. Specifically, two out of the total complications in the HD group occurred in the immediate postoperative period, including a deep SSI and a CSF leak, both graded as CDSC grade IIIb and IVb, respectively. On the other hand, the LD group experienced only late complications (21.4%), such as delayed infection and DJK.

This trend may be explained by HD constructs’ more significant surgical burden. The use of a higher number of screws, 22.7 in HD vs. 16.1 in LD (*p* = 0.001), can increase surgical time and risk of iatrogenic injury. As previously suggested in the literature, higher screw density correlates with elevated risk of neurologic, infectious, and dural complications [4,30].

Cost considerations remain central to surgical planning in EOS. Roach et al. emphasized that while modern constructs offer superior biomechanics, the clinical gains have not always matched the increase in cost [12]. Roach’s review underlined that outcomes from pedicle screw-only constructs are not necessarily superior to hybrid or lower-cost alternatives, which is particularly relevant in EOS patients who have already undergone costly distraction-based treatment, as shown in our findings, where HD constructs are related to significantly higher costs, with no apparent clinical advantage.

From a surgical strategy standpoint, this study supports a more tailored approach, especially in EOS patients previously treated with MCGRs. Even if HD constructs increase the implant cost, they give better outcomes in terms of early TK improvement and T1-S1 length. On the other hand, the frequent presence of auto-fusion, limited growth potential at the time of final fusion, and potential for retained fibrosis or metallosis may render full-density constructs unnecessary [28]. As shown in several of the articles reviewed, dual-rod constructs remain biomechanically favorable, but screw density per level can be modulated to reduce morbidity and cost.

This study fills a critical gap in the current literature by being the first to specifically examine the role of implant density in final fusion surgeries following MCGR treatment for EOS, and these findings offer valuable, targeted evidence to inform surgical planning, optimize outcomes, and reduce costs in a highly specialized and growing patient cohort.

### Limitations

The principal limitation of this study lies in its relatively small sample size, which significantly reduces the statistical power and the ability to detect meaningful differences, particularly concerning postoperative complications. A limited sample increases the risk of type II errors, thereby potentially obscuring clinically relevant associations. Furthermore, the small cohort may not be fully representative of the broader patient population, introducing the possibility of selection bias and limiting the generalizability of the findings. Finally, the restricted number of observations can magnify the influence of outliers, which may disproportionately affect the results and compromise the overall robustness of the study. Another limitation is associated with the retrospective design of the study, which may introduce information and selection biases, as well as limit the ability to control for potential confounding factors. Moreover, a potential confounding factor arises from the heterogeneous etiologies (idiopathic, syndromic, neuromuscular) and their varying distribution between the HD and LD groups. In fact, the different etiological distribution between the HD and LD groups—primarily idiopathic versus syndromic scoliosis, respectively—warrants careful consideration, as idiopathic patients generally have better bone quality and growth potential. These differences may affect implant density selection, complication rates, and radiographic outcomes. Furthermore, the decision regarding the type of ID to use was based exclusively on the clinical judgment and surgical experience of the operating surgeon, which is inherently subjective and may lead to variability in decision-making, contributing to a reduction in sample homogeneity. Additionally, the patients were not randomized, further limiting the ability to control for confounding factors and introducing the potential for selection bias. However, despite these limitations, the patients appeared to be comparable from a strictly radiographic perspective, with only the LL value showing a statistically significant difference at the end of lengthening. Furthermore, the follow-up duration was not identical between the HD and LD groups, potentially affecting complication detection. Additionally, no patient-reported outcome measures (PROMs) were considered in this study, which limits the ability to assess how ID might affect the quality of life of the patients examined. In conclusion, the absence of a formal power analysis, due to the retrospective nature of the study and the sample size being determined by available patient data rather than a predefined calculation, limits the ability to draw definitive conclusions about the effect size or the robustness of the findings.

Although this study did not account for these factors, it underscores the need for caution when extrapolating the results and emphasizes the importance of future research involving larger, stratified cohorts. Nonetheless, this study is the first to specifically address the issue of ID following MCGR lengthening and supports the emerging evidence that, when guided by sound biomechanical principles, lower ID can achieve outcomes comparable to those of HD constructs in spinal deformity surgery, including EOS treated with MCGRs.

## 5. Conclusions

This study is the first to evaluate the role of implant density in the final fusion of EOS patients previously treated with MCGR systems. Our findings indicate that while HD constructs provide slightly greater early postoperative kyphosis correction and spinal lengthening, they do not confer superior long-term radiographic or clinical outcomes compared to LD constructs. Moreover, high-density constructs were associated with a higher rate of severe early complications and significantly increased implant costs. Given the frequent presence of auto-fusion, curve rigidity, and reduced growth potential in this population, our data support a more individualized, biomechanically informed approach to screw placement. A lower implant density, when applied strategically, may achieve equally effective correction with fewer complications and lower economic impact.

## Figures and Tables

**Table 1:** Study variables.

Preoperative Data	Details
Sex	Male/female (M/F)
Age at surgery	Age (years)
BMI	Measured in kg/m^2^
Etiology of scoliosis	Type (AIS, NMS, SS)
Curve characteristic	Thoracic/double/lumbar
Main curve side	Right/left convex
Lumbar modifier	A/B/C
Follow-up	Follow-up time (months) between MCGR and final fusion and after final fusion
**Radiographic data**	
Main curve	Preoperatively, postoperatively, and at last follow-up (Cobb angle)
Percentage of curve correction	CR (%) for main curve
Frontal balance	The angle between the line connecting C7-S1 with respect to the vertical. Preoperatively, postoperatively, and at last follow-up.
Shoulder balance	The angle of the bi-coracoid line to the Horizontal. Preoperatively, postoperatively, and at last follow-up
Pelvis balance	The angle between the perpendicular to the line connecting the iliac wings and the line connecting T1-S1. Preoperatively, postoperatively, and at last follow-up
SVA	Measures the overall balance of the spine and corresponds to the horizontal distance between the plumb line of C7 and the posterosuperior corner of S1. Preoperatively, postoperatively, and at last follow-up
CL	Cobb angle between C2 and C7. Preoperatively, postoperatively, and at last follow-up
TK	Cobb angle between T5 and T12. Preoperatively, postoperatively, and at last follow-up
LL	Cobb angle between L1 and S1. Preoperatively, postoperatively, and at last follow-up
T1-T12 distance	Distance between the upper plate of T1 to upper plate of T12. Preoperatively, postoperatively, and at last follow-up
T1-S1 distance	Distance between the upper plate of T1 to upper plate of S1. Preoperatively, postoperatively, and at last follow-up
SS	The angle of the sacral plateau to the horizontal. Preoperatively, postoperatively, and at last follow-up
**Postoperative Data**	
UIV	UIV level (e.g., T2, T3, etc.)
LIV	LIV level (e.g., L3, L4, etc.)
Number of levels fused	Total levels fused (e.g., 10, 12, etc.)
Number of screws in construct	Total number of screws (n)
Density of the implant	Screw density (high density (HD) or low density (LD))
Cost of the implant	Calculated by adding the cost of the screws, rods, and cross-links in Euros (EUR)
Posterior column osteotomies performed	Number of cases with at least one posterior column osteotomy
**Postoperative clinical data**	
Duration of surgery	Total time of surgery (Minutes)
Blood loss	Total bleeding during surgery (mL)
Loss of Hb	Total points of Hb lost postoperatively (g/dL)
Complications	Type and number of perioperative complications (n, %) according to CDSC and type and number of late-onset complications (n, %)
LOS	Total duration of recovery (days)

AIS: Adolescent Idiopathic Scoliosis; NMS: Neuromuscular Scoliosis; SS: syndromic scoliosis; CDCS: Clavien–Dindo–Sink Classification; CR: correction rate; Hb: hemoglobin; LIV: Lower Instrumented Vertebra; LD: low-density; HD: high-density; SVA: Sagittal Vertical Axis; CL: Cervical Lordosis; TK: thoracic kyphosis; LL: Lumbar Lordosis; SS: Sacral Slope; UIV: Upper Instrumented Vertebra.

**Table 2:** Baseline variables.

Variable	HD (N = 13) (%)	LD (N = 14) (%)
**Sex**		
M	3 (23.1)	4 (28.5)
F	10 (76.9)	10 (71.5)
**Etiology**		
Idiopathic	9 (69.2)	4 (28.5)
Syndromic	2 (15.4)	8 (57.1)
Prader–Willi Syndrome		3 (21.4)
Sotos Syndrome		2 (14.2)
Cornelia De Lange Syndrome	1 (7.7)	
Robinow Syndrome		1 (7.1)
Coffin Syndrome		1 (7.1)
DiGeorge Syndrome		1 (7.1)
Larsen Syndrome	1 (7.7)	
Neuromuscular	2 (15.4)	2 (14.2)
Perinatal Asphyxia	2 (15.4)	
Congenital Myopathy		1 (7.1)
IIH		1 (7.1)
**Curve Characteristic**		
Thoracic	10 (76.9)	7 (50)
Double	2 (15.4)	6 (42.8)
Lumbar	1 (7.7)	1 (7.1)
**Main Curve Side**		
Right Convex	9 (69.2)	8 (57.1)
Left Convex	4 (30.6)	6 (42.8)
**Lumbar Modifier**		
A	7 (53.8)	7 (50)
B	4 (30.6)	4 (28.5)
C	2 (15.4)	3 (21.4)

IIH: Idiopathic Intracranial Hypertension.

**Table 3:** Demographic characteristics.

Parameters	HD (N = 13)	LD (N = 14)	*p*
Age (years, mean, SD, range)	14.2 (±1.2) (13–17)	14.5 (±1.6) (11–17)	0.483
Follow-up time between MCGR and final fusion (months, mean, SD, range)	60.1 (±25) (31–128)	66.5 (±29.6) (24–125)	0.748
Follow-up time after final fusion (months, mean, SD, range)	43.6 (±23.7) (24–96)	33.6 (±12.4) (24–60)	0.575
BMI (kg/m^2^, mean, SD, range)	21.3 (±3.5) (15.3–26.5)	22.5 (±7.5) (12.4–36.5)	0.572

BMI: Body Mass Index.

**Table 4:** Fixation characteristics.

Parameters	HD (N = 13) (%)	LD (N = 14) (%)	* p *
Number of pedicle screws (n, mean, SD, range)	22.7 (±4.5) (15–30)	16.1 (±3.9) (7–22)	** 0.001 **
Number of levels fused (n, mean, SD, range)	16.5 (±3) (11–21)	18.9 (±1.8) (15–21)	** 0.036 **
Number of pedicle screws per level (n, mean, SD, range)	1.38 (±0.2) (1.1–18)	0.8 (±0.2) (0.4–1.1)	** <0.001 **

**Table 5:** Osteotomies and UIV/LIV characteristics.

Variable	HD (N = 13) (%)	LD (N = 14) (%)
PCO (n, %)	6 (46.1)	1 (7.1)
UIV (n, %)		
T2	8 (61.5)	11 (78.5)
T3	4 (30.6)	2 (14.2)
T4	1 (7.7)	1 (7.1)
LIV (n, %)		
L2	3 (23.1)	2 (14.2)
L3	6 (46.1)	4 (28.6)
L4	4 (30.8)	8 (57.1)

LIV: Lower Instrumented Vertebra; PCO: posterior-column osteotomy; UIV: Upper Instrumented Vertebra.

**Table 6:** Frontal plane characteristics.

Variable	End of Lengthening (t1, Mean, SD, Range)			After Final Fusion (t2, Mean, SD, Range)			Last Follow-Up (t3, Mean, SD, Range)		
	HD	LD	* p *	HD	LD	* p *	HD	LD	* p *
** Main Curve (°) **	44.6 (±10.2) (25.5–65.9)	46.3 (±14.2) (23–73)	0.787	30.7 (±11.7) (9.6–49.1)	36 (±13.4) (8.4–62.4)	0.368	32.6 (±10.7) (15.2–50.2)	39.9 (±13.6) (14.2–64.7)	0.126
** Frontal Balance (°) **	2.4 (±2.1) (0.7–9.1)	4.2 (±4.3) (0.9–14.1)	0.435	4.3 (±5.4) (0.3–19.9)	4.7 (±4.5) (0.5–14.8)	0.465	2.9 (±2.9) (0.2–10.3)	3.2 (±4.7) (0.3–17.6)	0.412
** Shoulder Balance (°) **	2.8 (±1.9) (0.4–7.6)	5.4 (±5) (0.1–17.6)	0.254	3.8 (±2.4) (0.4–8.5)	4.6 (±4.5) (0.1–14.7)	0.984	5.1 (±4.4) (1.3–17.7)	4.3 (±5.1) (0.1–19.8)	0.368
** Pelvis Balance (°) **	2.7 (±2.1) (0.3–8.5)	4.2 (±3.5) (0.1–12.8)	0.207	3.5 (±4.1) (0.5–16)	3.8 (±2.9) (0.9–9.6)	0.412	2.8 (±2.8) (0.1–11.1)	3.2 (±2.8) (0.1–8.6)	0.849

**Table 7:** Sagittal plane characteristics.

Variable	End of Lengthening (t1, Mean, SD, Range)			After Final Fusion (t2, Mean, SD, Range)			Last Follow-Up (t3, Mean, SD, Range)		
	HD	LD	* p *	HD	LD	* p *	HD	LD	* p *
** SVA (mm) **	−13.4 (±54.9) (−111.3–79.6)	5.9 (±42.6) (−86.4–78.9)	0.453	7.6 (±62.1) (−81.2–169.8)	2.6 (±31) (−50.2–67.7)	0.984	3.8 (±52.3) (−79.3–88.6)	24.8 (±41.3) (−26.8–99.5)	0.509
** LL (°) **	56.2 (±14.2) (38.5–82.3)	42.8 (±14.9) (22.9–74.9)	** 0.023 **	45.8 (±16.6) (23.9–76.7)	46.7 (±14.1) (26.6–82)	0.825	42.4 (±20.8) (2.2–71.2)	46.9 (±18) (15.4–82.1)	0.718
** TK (°) **	23.4 (±13.8) (8.3–62.2)	16.5 (±18.9) (−23.7–55.2)	0.197	16.3 (±7.6) (8.6–38.7)	10.9 (±14.4) (−14.1–48.9)	** 0.021 **	15.8 (±9.5) (2.7–32.4)	13.7 (±18.4) (−13.1–62.7)	0.293
** CL (°) **	9.4 (±28.2) (−25–83.2)	−0.1 (±14.2) (−21.8–27)	0.541	7.4 (±25.6) (−28.8–67.9)	−7.2 (±22.7) (−31.6–45.3)	0.767	9.5 (±25.1) (−24.9–62.7)	−0.3 (±21) (−33.5–51.1)	0.509
** SS (°) **	38.1 (±9.5) (23.1–52.6)	32.8 (±10.5) (17.7–54.4)	0.254	35.1 (±12.1) (17.6–57.1)	38.1 (±12.3) (19.6–65.4)	0.509	35.9 (±11.3) (19.9–58.2)	38.2 (±10.9) (18.−61.2)	0.596
** T1-T12 (mm) **	22.5 (±2.2) (26.2–19.9)	21.8 (±3.3) (13.9–29.5)	0.624	24.2 (±5.6) (15.5–40.7)	21.9 (±3.7) (14.7–29.6)	0.167	26.2 (±5.9) (17.8–42.6)	22.7 (±3.9) (14.3–28.7)	0.126
** T1-S1 (mm) **	39.8 (±3.6) (35.6–46)	37.5 (±3.8) (27.7–44.7)	0.214	43.3 (±6.7) (31.1–59.1)	39.1 (±4.3) (29.5–46.9)	0.039 *	45.4 (±7) (33.4–62.3)	39.7 (±5.1) (28.5–47.5)	0.021 *

CL: Cervical Lordosis; LL: Lumbar Lordosis; SS: Sacral Slope; SVA: Sagittal Vertical Axis; TK: thoracic kyphosis. * *p* < 0.05.

**Table 8:** Surgical outcomes.

Parameters	HD (N = 13)	LD (N = 14)	* p *
Cost of the implant (EUR, mean, SD, range)	6046.5 (±1146.9) (4085–7910)	4376.4 (±999.4) (2045–5870)	** <0.001 **
Surgery duration (min, mean, SD, range)	268.1 (±46.1) (202–336)	241.2 (±28.1) (195–290)	0.108
Loss of Hb (g/dL, mean, SD, range)	1.4 (±0.7) (0.1–2.4)	1.6 (±0.8) (0.2–3.2)	0.453
Blood loss (mL, mean, SD, range)	630.7 (±222.2) (300–1100)	521.4 (±279.9) (200–1300)	0.138
LOS (days, mean, SD, range)	8.3 (±1.8) (6–13)	8.7 (±1.7) (6–13)	0.370

Hb: hemoglobin; LOS: length of hospital stay.

**Table 9:** Postoperative complications.

Variable	HD (N = 13) (%)	LD (N = 14) (%)
Total complications (n, %)	3 (23.1)	3 (21.4)
Perioperative complications (n, %)	2 (15.4)	0
Deep SSI	1 (7.7)	/
CSF leak	1 (7.7)	/
CDSC	/	/
Grade I	/	/
Grade II	/	/
Grade IIIa	/	/
Grade IIIb	1 (7.7)	/
Grade IVa	/	/
Grade IVb	1 (7.7)	/
Grade V	/	/
Late-onset complications (n, %)	1 (7.7)	3 (21.4)
Delayed SSI	/	1 (7.1)
Rod rupture	1 (7.7)	/
DJK	/	2 (14.2)

CDSC: Clavien–Dindo–Sink Classification; CSF: cerebrospinal fluid, DJK: distal junctional kyphosis; SSI: surgical site infection.

## Data Availability

The datasets used and/or analyzed during the current study are not publicly available due to our policy statement of sharing clinical data only on request but are available from the corresponding author on reasonable request.

## Abbreviations

AIS	Adolescent Idiopathic Scoliosis
AP	Anteroposterior
AS	All-screw
BMI	Body Mass Index
CDC	Clavien Dindo Classification
CDCS	Centers for Disease Control and Prevention Scale
CR	Correction Rate
DSI	Delayed Surgical Site iInfection
EOS	Early-onset Scoliosis
FUP	Follow-up
HD	High-density
HC	Hybrid Constructs
Hb	Hemoglobin
ICU	Intensive Care Unit
LD	Low-density
LIV	Lower Instrumented Vertebra
LL	Lumbar Lordosis
LOS	Length of Hospital Stay
MCGR	Magnetically Controlled Growing Rods
MEPs	Motor-evoked Potentials
PCOs	Posterior Column Osteotomies
PROMs	Patient-Reported Outcome Measures
SEPs	Sensory-evoked Potentials
SSI	Superficial Site Infection
STROBE	Strengthening the Reporting of Observational Studies in Epidemiology
TGR	Traditional Growing Rods
TK	Thoracic Kyphosis
UIV	Upper Instrumented Vertebra
